# Community Treatment Centers for Isolation of Asymptomatic and Mildly Symptomatic Patients with Coronavirus Disease, South Korea

**DOI:** 10.3201/eid2610.201539

**Published:** 2020-10

**Authors:** Won Suk Choi, Hyoung Seop Kim, Bongyoung Kim, Soomin Nam, Jang Wook Sohn

**Affiliations:** Korea University, Seoul, South Korea (W.S. Choi, J.W. Sohn);; National Health Insurance Ilsan Hospital, Goyang, South Korea (H.S. Kim, S. Nam);; Hanyang University, Seoul (B. Kim)

**Keywords:** respiratory infections, severe acute respiratory syndrome coronavirus 2, SARS-CoV-2, SARS, COVID-19, 2019 novel coronavirus disease, coronavirus disease, zoonoses, viruses, coronavirus, extended care facilities, isolation, South Korea

## Abstract

As a part of measures to decrease spikes in coronavirus disease (COVID-19) cases and deaths outside of hospitals, the government of South Korea introduced a plan for community treatment centers (CTCs) to isolate and monitor patients with mild COVID-19 symptoms. We assessed outcomes of 568 patients admitted to 3 CTCs near Daegu. More (64.6%) women than men (35.4%) were admitted, and the mean age of patients was 36.0 years (SD +15.0 years). Among all patients, 75.7% remained asymptomatic while at the CTCs. The mean time patients remained at CTCs was 19.6 days (SD +5.8 days) from the day of diagnosis until our study ended on March 23, 2020. Because they offer appropriate clinical triaging and daily monitoring for patients, CTCs are a safe alternative to medical institutions for asymptomatic or mildly symptomatic patients with COVID-19.

Since initial reports of coronavirus disease (COVID-19) from Wuhan, China, 267,013 confirmed COVID-19 cases have been reported from 184 countries, as of March 22, 2020 (*1*). In South Korea, severe acute respiratory syndrome coronavirus 2 (SARS-CoV-2), which causes COVID-19, was detected in a person from China who entered the country from Wuhan on January 19, 2020 (*2*). After an outbreak was identified among a religious group in Daegu and the neighboring regions on February 18, 2020, the cumulative number of cases in South Korea increased dramatically (*3*). Because of the sharp increase in cases in this region, it was impossible to accommodate all patients in hospitals. The shortage of hospital beds left >2,000 persons with confirmed COVID-19 waiting many days at home for a hospital admission. Unfortunately, several persons died at home while waiting or during transportation to the hospital. As a part of measures to decrease spikes in COVID-19 caseloads in and deaths outside of hospitals, the government of South Korea converted private dormitories and state-run institutions into community-based isolation facilities for patients with laboratory-confirmed COVID-19, but mild or no symptoms. These community treatment centers (CTCs) enabled the efficient use of medical institutions and compensated for the shortcomings of self-isolation. South Korea opened its first CTC on March 2, 2020, and by March 19, 2020, 16 CTCs with a total of 3,818 beds were distributed across the country. We describe the operating processes of 3 CTCs near Daegu, South Korea, and analyze the clinical characteristics and disease progression in admitted patients.

## Materials and Methods

### Participating Community Treatment Centers

**Figure 1:**
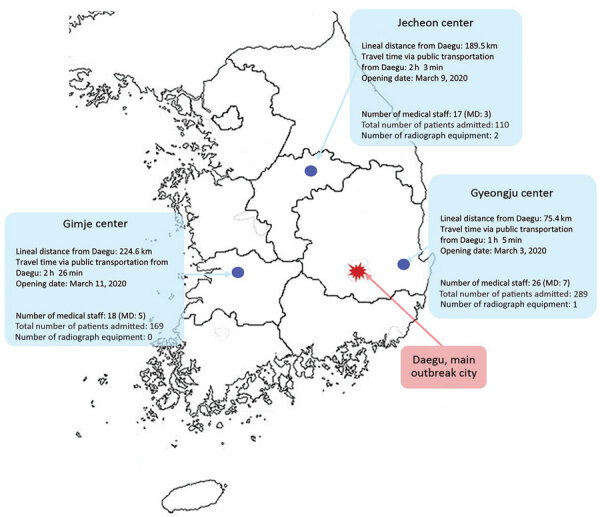
Geographic distribution of participating community treatment centers for isolation of mildly symptomatic and asymptomatic persons with diagnosed coronavirus disease, South Korea. MD, medical doctor.

The 3 CTCs that participated in this study each had a capacity to house 136–235 patients (Figure 1). All patients were from Daegu, where a large outbreak occurred, and tested positive for SARS-CoV-2 by real-time reverse transcription PCR (rRT-PCR) assays of upper respiratory tract (nasal and pharyngeal) or lower respiratory tract (sputum) specimens. Patients admitted to CTCs were classified as having mild or asymptomatic COVID-19 by epidemiologic investigators in Daegu. According to Korea Centers for Disease Control and Prevention (KCDC) guidelines (*4*), asymptomatic patients were alert, <50 years of age, nonsmokers who had no concurrent conditions and body temperature <37.5°C without antipyretic drugs. Patients with mild disease were alert and met >1 of the following criteria: age <50 years, no concurrent conditions, and body temperature <38°C with antipyretics (*4*). Patients were admitted to CTCs because they could not self-isolate at home for medical or nonmedical reasons, including impaired performance of daily activities and unfeasibility of home isolation. Children were admitted and most were in infected family groups who were housed together in the centers. Patients with laboratory-confirmed COVID-19 who met at >1 of the following criteria were considered severe cases and were hospitalized immediately for treatment: >65 years of age; >1 underlying condition, such as diabetes, chronic kidney disease, chronic liver disease, chronic pulmonary disease, chronic cardiovascular disease, hematologic malignancy, undergoing chemotherapy, or use of immunosuppressants; required oxygen therapy; or needed special care, including persons who were severely obese, pregnant, or required renal dialysis (*4*).

Candidates for CTC admission arrived at the centers from their homes by designated buses offered by the Daegu local government. The buildings in all CTCs were divided into clean and contaminated zones. The clean zone was the working and living space designated for staff and the contaminated zone was the isolation space designated for patients. When entering the contaminated zone all staff were required to wear personal protective equipment, including N95 respirators, gloves, goggles, and hooded coveralls.

The 3 CTCs opened on different days; Gyeongju on March 3, Jecheon on March 9, and Gimje on March 11. Each CTC was paired with a large hospital that coordinated and established operations and dispatched medical staff, including 1 physician and 1 nurse per CTC, and other necessary staff. The Gyeongju CTC had 1 radiograph unit and the Jecheon CTC had 2 radiograph units; Gimje CTC did not have an radiograph unit (Table 1). In addition to the medical professionals from private hospitals, the Gimje and Gyeongju CTCs included army physicians, public health physicians, and volunteer nurses, recruited for system operations. The Jecheon CTC was operated solely by medical professionals dispatched from a public hospital. Medical professionals stationed at each CTC monitored patients’ conditions, collected patient specimens for rRT-PCR, and were on hand for emergencies requiring hospital transfer.

**Table 1:** Characteristics of 3 community treatment centers, South Korea

Characteristics	Gimje	Gyeongju	Jecheon
Patient capacity	210	235	136
Opening date	2020 Mar 11	2020 Mar 3	2020 Mar 9
Matching hospital	Hanyang University Seoul Hospital	Korea University Medical Center	National Health Insurance Service Ilsan Hospital
No. medical staff			
Doctors, public sector*	4	6	3
Doctors, private sector	1	1	0
Registered nurses	7	9	6
Assistant nurses	6	9	2
Other†	0	1	6
No. staff from other sectors			
Local government	10	10	8
Central government, including the Ministry of Health and Welfare	3	3	2
Facilities management	6	6	16
Disinfection	10	9	11
Military	10	8	4
Police	6	8	6
Fire	1	1	1
No. radiography units	0	1	2


*Includes public health doctors, army doctors, and doctors from a public hospital. †Includes radiologic technicians, physical therapists, occupational therapists.

Apart from healthcare professionals, Daegu local government, in cooperation with the central government, primarily managed CTCs and provided administrative support, including providing medical equipment and meals. In addition, personnel from the military, police, and fire departments were stationed at the CTCs to provide operational services, including food delivery, access control, and patient transfer in emergencies. Each CTC required 64–72 personnel per day to maintain operations.

### Discharge Criteria

Discharge decisions were based on rRT-PCR assays of nasopharyngeal or sputum specimens to detect SARS-CoV-2 (*5*). Green Cross Laboratories (https://www.gclabs.co.kr) performed rRT-PCR for all 3 CTCs by using Allplex 2019-nCoV assays (Seegene Medical Foundation, https://www.seegenetech.com). KCDC set discharge guidelines, which required negative results for 2 serial rRT-PCR tests performed >24 hours apart (*6*).

### Monitoring and Testing Processes

During isolation in the CTCs, patients had their temperatures and respiratory symptoms checked >2 times each day, either by medical staff or by using self-monitoring equipment. Medical staff determined whether chest radiography or measurement of oxygen saturation were needed at admission, worsening of symptoms, or discharge. Each CTC had medications for symptomatic treatment, such as antipyretics and antitussives, which were prescribed by the medical staff. Each center had a portable oxygen tank if needed.

**Figure 2:**
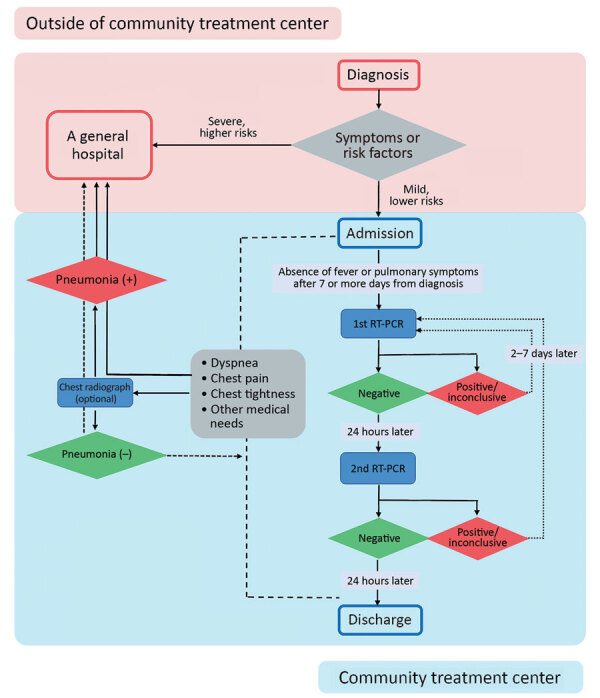
Flowchart demonstrating assessment before admission to community treatment centers, real-time reverse transcription PCR testing, and discharge process for mildly symptomatic and asymptomatic patients with diagnosed coronavirus disease, South Korea. RT-PCR, reverse transcription PCR.

For patients with no fever, pulmonary symptoms, or use of antipyretics, an rRT-PCR test was performed >7 days from the day of diagnosis. Subsequent rRT-PCR tests were performed >24 hours later if the initial result was negative or in 2–7 days if the initial result was positive or inconclusive. Patients who developed symptoms such as dyspnea, chest pain, or chest tightness or had abnormal findings suggesting pneumonia on chest radiographs were transferred to a hospital. Patients were discharged when they met the rRT-PCR testing requirements provided by KCDC (Figure 2).

### Data Collection

We used CTC records to collect data on patients from the day of admission to March 22, 2020. Basic medical information was collected by CTC staff through a web-based questionnaire or a telephone interview at the time of admission. Patients were asked the date of symptom onset, the date of COVID-19 diagnosis, whether they had underlying conditions, and whether they had symptoms associated with COVID-19 (Appendix). A questionnaire for daily health self-monitoring was distributed 2 times a day and asked for self-monitored temperatures, whether patients had symptoms associated with COVID-19, and whether they had other healthcare-related questions (Appendix). The Gimje and Jecheon CTCs used text messaging to distribute links to questionnaires that were refined by using a Google survey platform (https://www.google.com). The Gyeongju CTC used a personal health record-based real-time monitoring system (Softnet, https://www.softnet.co.kr) and provided instructions to the patients at admission; staff called patients who did not complete the questionnaire on time.

### Statistical Analysis

To analyze clinical characteristics of patients with persistent detection of SARS-CoV-2 by rRT-PCR for >28 days, we excluded patients who met the following criteria from the analysis: patients staying at the center on the 28th day from the day of initial diagnosis; and patients with no rRT-PCR test results or only 1 negative rRT-PCR test result performed after the 28th day from the day of initial diagnosis. We conducted all statistical analyses by using SPSS Statistics 24.0 for Windows (IBM Corp., https://www.ibm.com). We analyzed categorical variables by using the χ^2^ test or Fisher exact test, as appropriate. We analyzed continuous variables by using independent *t* tests and considered 2-tailed p<0.05 statistically significant.

The study protocol was approved by the Institutional Review Board (IRB) of Korea University Ansan Hospital, Seoul (IRB no. 2020AS0083). The requirement for written informed consent from patients was waived due to the nature of the study and unfeasibility related to the same.

## Results

### Clinical Characteristics of Patients

By March 23, 2020, a total of 568 patients had been admitted to the 3 CTCs: Gimje admitted 169 (29.7%), Gyeongju admitted 289 (50.9%), and Jecheon admitted 110 (19.4%). At the end of the study period, 356 (62.7%) patients remained in the centers, 200 (35.2%) had returned home and into society, and 12 (2.1%) were transferred to hospitals for further treatment (Table 2).

**Table 2:** Clinical characteristics of 568 patients with no or mild symptoms of coronavirus disease isolated 3 in community treatment centers, South Korea*

Characteristics	Total, n = 568	Gimje, n = 169	Gyeongju, n = 289	Jecheon, n = 110
Current statistics				
In isolation in community treatment center	356 (62.7)	131 (77.5)	147 (50.9)	78 (70.9)
Discharged with recovery	200 (35.2)	33 (19.5)	137 (47.4)	30 (27.3)
Transferred to a hospital	12 (2.1)	5 (3.0)	5 (1.7)	2 (1.8)
Sex				
F	367 (64.6)	101 (59.8)	185 (64.0)2	81 (73.6)
M	201 (35.4)	68 (40.2)	104 (36.0)	29 (26.4)
Age, mean ±SD	36.0 ± 15.0	33.4 ± 14.6	37.8 ± 14.5	35.0 ± 16.2
Underlying conditions†	36 (6.3)	3 (1.8)	26 (9.0)	7 (6.4)
COVID-19 symptoms over the course of disease‡				
N	430 (75.7)	115 (68.0)	238 (82.4)	77 (70.0)
Y	138 (24.3)	54 (32.0)	51 (17.6)	33 (30.0)
rRT-PCR tests per patient, mean ±SD	2.83 ± 1.17	2.82 ± 1.04	2.73 ± 1.26	3.11 ± 1.09
rRT-PCR tests needed before discharge criteria met, % patients§				
2	33.3 (189/568)	23.7 (40/169)	43.3 (125/289)	21.8 (24/110)
3	12.4 (47/379)	17.8 (23/129)	11.6 (19/164)	5.8 (5/86)
4	14.5 (48/332)	7.5 (8/106)	19.3 (28/145)	14.8 (12/81)
rRT-PCR results				
Follow-up 1	N = 558	N = 166	N = 284	N = 108
Negative	307 (55.0)	65 (39.2)	188 (66.2)	54 (50.0)
Positive	143 (25.6)	56 (33.7)	58 (20.4)	29 (26.9)
Inconclusive	108 (19.4)	45 (27.1)	38 (13.4)	25 (23.1)
Follow-up 2	N = 539	N = 164	N = 267	N = 108
Negative	295 (54.7)	85 (51.8)	172 (64.4)	38 (35.2)
Positive	119 (22.1)	32 (19.5)	50 (18.7)	37 (34.3)
Inconclusive	125 (23.2)	47 (28.7)	45 (16.9)	33 (30.5)
Follow-up 3	N = 292	N = 96	N = 123	N = 73
Negative	14 (49.0)	42 (43.8)	72 (58.5)	29 (39.7)
Positive	53 (18.1)	20 (20.8)	16 (13.0)	17 (23.3)
Inconclusive	96 (32.9)	34 (35.4)	35 (28.5)	27 (37.0)
Follow-up 4	N = 152	N = 33	N = 74	N = 45
Negative	81 (53.3)	20 (60.6)	39 (52.7)	22 (48.9)
Positive	202 (13.2)	1 (3.0)	12 (16.2)	7 (15.5)
Inconclusive	51 (33.5)	12 (36.4)	23 (31.1)	16 (35.6)
Days in isolation, mean ±SD¶				
All patients, 2020 Mar 23	19.6 ± 5.8	17.9 ± 5.2	21.3 ± 5.9	17.9 ± 5.5
Patients currently admitted	22.2 ± 5.0	19.1 ± 5.0	25.9 ± 3.4	20.7 ± 2.6
Patients discharged with recovery	15.6 ± 4.0	14.5 ± 3.7	16.7 ± 3.6	11.6 ± 3.5
Patients transferred to a hospital	9.6 ± 5.2	11.0 ± 3.7	11.4 ± 4.4	1.5 ± 2.1

*As of March 23, 2020. Values are no. (%) except where otherwise indicated. CTC, community treatment center; rRT-PCR, real-time reverse transcription PCR. †Includes any chronic disease requiring medication, such as diabetes or hypertension. ‡Includes fever, dyspnea, cough, sputum, nasal congestion, decreased sense of smell or taste, sore throat, or diarrhea. §Two negative results >24 h apart are required before patient discharge. ¶Includes the period of self-isolation at home before being admitted at the center.

More women (64.6%) were admitted than men (35.4%), and the mean age of patients was 36.0 years (SD +15.0 years). A small proportion (6.3%) of patients had >1 chronic disease requiring medication, such as diabetes and hypertension. Many (75.7%) remained asymptomatic over the course of the disease, but 138 (24.3%) reported symptoms associated with COVID-19. The most common symptoms were cough (11.6%) and nasal congestion (9.8%).

The mean number of rRT-PCR tests performed for each patient was 2.83 (SD +1.17), and 33.3% (189/568) of patients were released from isolation at the 2nd follow-up test. Of the patients remaining in the CTCs, 12.4% (47/379) were released after the 3rd follow-up test and 14.5% (48/332) after the 4th. Among the first follow-up rRT-PCR tests, which marked the beginning of the discharge process, 55.0% were negative, 25.6% positive, and 19.4% inconclusive. The proportion of positive results showed a decreasing trend, but inconclusive results showed an increasing trend (Table 2).

The mean number of days patients remained at the CTCs from the date of initial diagnosis until discharge or the end of the study period on March 23, 2020, was 19.6 (SD +5.8). For discharged patients, the mean number of days between diagnosis and discharge was 15.6 (SD +4.0). The mean number of days between COVID-19 diagnosis and transfer of a patient to the hospital was 9.6 (SD +5.2).

### Clinical Characteristics of Patients with Persistent Viral Detection >28 days

A total of 19 patients had positive or inconclusive rRT-PCR results >28 days after initial diagnosis. Among them, 78.9% were female, 22.1% were male, the mean age was 38.4 years (SD +13.6 years), 5.3% had underlying conditions, and 15.8% had COVID-19 symptoms. No statistically significant differences in overall clinical characteristics were noted between patients with persistent detection of virus ≥28 days and others. Additional rRT-PCR tests (mean 4.05, SD +1.08) were performed for patients with persistent viral detection compared with those who were discharged <28 days after diagnosis (mean 2.76, SD +1.10; p<0.001) (Table 3).

**Table 3:** Characteristics of 337 asymptomatic or mildly symptomatic patients with prolonged detection of severe acute respiratory syndrome coronavirus 2 admitted to community treatment centers for isolation, South Korea*

Center	Positive rRT-PCR >28 d, no. (%)	Release from isolation ≤28 d, no. (%)	p value
Gimje	1 (5.3)	83 (26.1)	0.077
Gyeongju	16 (84.2)	189 (59.4)	ND
Jecheon	2 (1.1)	46 (14.5)	ND
Sex			
F	15 (78.9)	202 (63.5)	0.173
M	4 (21.1)	116 (36.5)	Referent
Mean age, y, ±SD	38.4 ± 13.6	36.5 ± 15.4	0.595
Presence of underlying conditions†	1 (5.3)	27 (8.5)	1.000
Presence of signs and symptoms‡	3 (15.8)	63 (19.8)	1.000
No. rRT-PCR tests, mean ±SD	4.05 ± 1.08	2.76 ± 1.10	<0.001

*ND, not done; rRT-PCR, real-time reverse transcription PCR. †Includes any chronic underlying condition requiring medication, such as diabetes or hypertension. ‡Includes fever, dyspnea, cough, sputum, nasal congestion, decreased sense of smell or taste, sore throat, or diarrhea.

### Clinical Characteristics of Patients Transferred to Hospitals

A total of 12 patients were transferred to hospitals; 5 each from Gimje and Gyeongju and 2 from Jecheon. The median age of patients transferred to a hospital was 43.5 years (interquartile range [IQR] 34.25–60.25 years), and 58.3% were women. Three (25.0%) patients had underlying conditions, including schizophrenia, hypertension, and diabetes. Eight (66.7%) patients were transferred with symptoms suggesting aggravated COVID-19; 2 were transferred with medical issues not associated with COVID-19; 2 were transferred for special care, including a 2-year-old who was too young to be taken care of at a CTC and a pregnant woman. One patient was transferred for personal reasons. The median number of days from admission to hospital transfer was 2.5 days (IQR 2.0–6.75 days) (Table 4).

**Table 4:** Clinical characteristics of 12 patients with coronavirus disease transferred from community treatment centers to a hospital, South Korea*

Age, y/sex	Center	Underlying conditions	Reason for transfer	Symptoms and signs suggesting pneumonia at transfer			No. days from admission to transfer
				Fever, temperature ≥37.5°C	Desaturation, SpO_2_ <95	Abnormal findings on chest radiograph	
56/F	Gimje	No	Dyspnea	No	No	NA	1
42/F	Gimje	No	Cough, chest tightness	No	No	NA	2
42/M	Gimje	No	Purulent otorrhea	No	No	NA	3
45/M	Gimje	No	Dyspnea	No	No	NA	6
38/F	Gimje		Personal issue†	No	No	NA	10
27/M	Gyeongju	Schizophrenia	Aggravation of schizophrenia	No	No	NA	14
65/F	Gyeongju	Hypertension	Fever	Yes	No	Yes	2
58/M	Gyeongju	Diabetes mellitus, hypertension	Dyspnea	No	Yes	NA	2
2/F	Gyeongju	No	Need for special care‡	No	No	NA	2
33/F	Gyeongju	No	Need for special care§	No	No	NA	7
61/F	Jecheon	No	Dyspnea	No	No	Yes	3
65/M	Jecheon	No	Dyspnea, pleuritic pain	No	No	Yes	1

*NA, not applicable. †Patient’s child admitted to hospital with confirmed coronavirus disease during her admission; she asked to transfer to the hospital where her child was admitted. ‡Patient too young to be in the center without parents. §Patient 9 weeks pregnant at admission.

## Discussion

Our experience illustrates that CTC operations can be a safe alternative to conventional medical institutions. South Korea introduced CTCs to cope with the rapidly growing number of patients with COVID-19 who required isolation and monitoring but did not necessarily need to be hospitalized for treatment. Patients admitted to CTCs maintained a stable clinical course, but the time to discharge was long.

Isolation facilities for mild cases were vital to helping overcome COVID-19 outbreaks in the country, particularly because >80% of cases were not severe and did not require special therapies, such as oxygen supplementation or parenteral fluid infusion (*7*). Introducing CTCs effectively ensured that hospital beds were available for patients with moderate or severe disease. In Daegu, during the first phase of the outbreak, some patients likely died due to the unavailability of hospital beds (*8*), and increasing admissions could have led to the collapse of the healthcare system. Because of several timely countermeasures, including the rapid establishment of CTCs, the mortality rate for COVID-19 in South Korea remained lower (2.4%) than in other countries, including the United States, 6.0%; Japan, 4.3%; China, 5.6%; Iran, 6.0%; and Italy, 14.1% (*9*). In addition, CTCs helped curb virus transmission in the population. Although violation of the self-isolation orders in South Korea is punishable by law, some cases of nonadherence have been witnessed (*10*).

The KCDC patient classification system for COVID-19 severity was essential for operating the CTCs. As part of city- and province-level patient management teams, epidemiologic investigators classified all confirmed cases by severity and ensured patients with severe symptoms were hospitalized and that other patients received appropriate treatment options (*6*). For patients without severe disease, epidemiologic investigators decided whether to send them to a hospital or a CTC on the basis of hospital bed capacity. Because hospital beds were unavailable in the middle of the outbreak, some patients admitted to CTCs did not meet the criteria of mild disease precisely. In our study, 42 patients were not classified accurately and should have been hospitalized instead of admitted to CTCs. Of them, 6 patients experienced intensified symptoms and were transferred to hospitals (data not shown). Such misclassification can be attributed to the urgent situation in Daegu and the surrounding areas and the unfamiliarity with the novel patient classification system. Fortunately, misclassifications decreased over time.

Most patients with COVID-19 admitted to CTCs were asymptomatic or had only mild symptoms over the course of the disease. Patients who were discharged from the hospitals but still had positive viral detection could be admitted to CTCs, but we did not have any patients of this demographic in our study.

Of note, ≈90% of patients were asymptomatic at the time of admission (data not shown). Extensive and aggressive testing was performed on close contacts of SARS-CoV-2–infected patients in Daegu, especially among members of a specific religious group in which a large outbreak occurred, which possibly contributed to the exceptionally high proportion of asymptomatic cases. Another finding of note was that 5.6% (19/337) of patients had positive or inconclusive rRT-PCR test results, even >28 days after diagnosis, which could indicate that viral shedding continues longer than assumed. A study of 56 patients with mild to moderate COVID-19 symptoms indicated that the median duration of viral shedding was 24 days, and the longest was 42 days (*11*). Data from another study of 137 patients showed that the median duration of viral detection was 12 days, and the maximum was 45 days (*12*). However, viral RNA detection does not imply infectivity. According to a report from the US Centers for Disease Control and Prevention, when viral RNA in upper respiratory samples was continuously detected in a patient following clinical recovery, the RNA concentration was generally below the level at which replication-competent virus can be isolated reliably (*13*).

Our study has several limitations. First, data on patients, especially those who were still in the CTCs at the end of the study, did not reflect the complete clinical course, and we were not able to evaluate the time between the diagnosis and discharge for all patients. Of note, observation of the entire clinical course of patients was not possible because some CTCs closed and patients were transferred to other centers as the outbreak was stabilized; for instance, Jecheon closed on April 3, Gimje on April 7, and Gyeongju on April 14. Operation of all CTCs that opened for the outbreak in Daegu and surrounding areas ended on April 30, 2020. Because the COVID-19 pandemic continues, we decided to present the data collected up to March 23 to provide information on CTCs and the clinical characteristics of patients with mild disease. Second, because of the evolving emergency, protocols for patient care varied slightly among centers and a standardized protocol still does not exist. A standardized protocol for patient care that includes the discharge process and transfer criteria should be developed in preparation for a potential second wave of the pandemic. Finally, data collection for clinical symptoms and other medical conditions was dependent on web- or application-based questionnaires and the information obtained might be exaggerated or underestimated. To compensate for this, direct communication or telecommunication was used in extraordinary situations and for those who failed to respond to questionnaires; the response rate was >80% in each center.

In conclusion, 75.7% of patients admitted to CTCs in South Korea were asymptomatic, and most maintained a stable clinical course until discharge. Appropriate clinical triaging and CTC operations that include daily patient monitoring are a safe alternative to medical institutions for asymptomatic and mildly symptomatic patients diagnosed with COVID-19 during a pandemic.

Appendix Questionnaires used for daily monitoring of asymptomatic and mildly symptomatic patients with coronavirus disease admitted to community treatment centers for isolation, South Korea.
