# Supplementary material for: Community Treatment Centers for Isolation of Asymptomatic and Mildly Symptomatic Patients with Coronavirus Disease, South Korea
Source: Emerg Infect Dis. 2020 Oct;26(10):2338–45. doi: 10.3201/eid2610.201539 (PMC7510708; doi:10.3201/eid2610.201539)
Supplement: Appendix — Questionnaires used for daily monitoring of asymptomatic and mildly symptomatic patients with coronavirus disease admitted to community treatment centers for isolation, South Korea. [file 20-1539-Techapp-s1.pdf]

Article DOI: https://doi.org/10.3201/eid2610.201539

Community Treatment Centers for Isolation of Asymptomatic and Mildly Symptomatic Patients with Coronavirus Disease, South Korea

Appendix

Questionnaire for Basic Medical Information

A. Gimje Center

1. Name: ____________
2. Location
   - □ Building B
   - □ Building C
3. Room number:
4. Did you have following symptoms?
   - □ No symptom
   - □ Fever (≥37.5°C)
   - □ Coughing
   - □ Muscle pain
   - □ Runny nose or stuffy nose
   - □ Sore throat
   - □ Dyspnea
   - □ Feebleness (Malaise)
   - □ Diarrhea
   - □ Abdominal pain
   - □ Others (____________)
5. What is the date of initial symptom expression? (If you don't have any symptoms, please skip this question)
   __/___/___ (Day/Month/Year)
6. What is the date of diagnosis of COVID-19
   __/___/___ (Day/Month/Year)

7. Underlying conditions (Please check all the disease you have.)
 Hypertension
 Diabetes mellitus
 Chronic Renal disease
 Chronic liver disease
 Chronic pulmonary disease
 Chronic cardiovascular disease (except hypertension)
 Hematologic malignancy
 Undergoing chemotherapy
 Use of immunosuppressants
 HIV
 Others (____________)

8. Any of following?
 Obesity
 Pregnant
 Transplantation recipient
 Others (____________)
 Not applicable

B. Gyeongju Center

1. Name: ____________
2. Sex
 Male
 Female
3. Date of birth: ___/____/____ (Day/Month/Year)
4. What is the date of initial symptom expression? (If you don't have any symptoms, please skip this question)
__/___/___ (Day/Month/Year)
5. Underlying conditions: ____________
6. Are you pregnant?
 Yes
 No
7. Did you have following symptoms
 Fever (≥37.5°C)
 Coughing
 Muscle pain
 Runny nose or stuffy nose
 Sore throat
 Dyspnea
 Feebleness (Malaise)
 Diarrhea
 Abdominal pain
 Others (____________)
8. Has the medication been taken in the last 24 hours?
 Yes (____________)
 No
9. Others: ____________

C. Jecheon Center

1. Name: ____________
2. Sex
   - Male
   - Female
3. Date of birth: ___/____/____ (Day/Month/Year)
4. Location
   - Building B
   - Building C
5. Room number: ____________
6. Mobile phone number: ____________
7. Mobile phone number of guardians: ____________
8. Underlying disease (Please check all the disease you have.)
   - Hypertension
   - Diabetes mellitus
   - Renal disease
   - Others (____________)
   - None
9. Do you have sufficient medication more than 1 month?
   - Yes (Sufficient)
   - No (Not sufficient)
   - Irrelevant
10. If you don’t have sufficient medication, do you have supplement plan?
    - I have family member who can deliver medication
    - I need someone to help purchase medication for me
    - Irrelevant
11. Are you pregnant now?
    - No
    - Yes
    - Irrelevant
    - Others (____________)
12. Clinical symptoms (Please check all the symptoms you have.)
    - No symptom
    - Fever (≥37.5°C)
    - Coughing
    - Muscle pain
    - Runny nose or stuffy nose
    - Sore throat
    - Dyspnea
    - Feebleness (Malaise)
    - Diarrhea
    - Abdominal pain
    - Others (____________)

13. Medication you had during 24 hours (Please check all the drugs you had)
    - ☐ General medication for cold
    - ☐ Fever remedy
    - ☐ Antibiotics
    - ☐ Medications for hypertension
    - ☐ Medications for diabetes
    - ☐ None
    - ☐ Others (____________)

Questionnaire for Daily Health Self-Monitoring

A. Gimje Center

1. Name: ____________
2. Location
    - ☐ Building B
    - ☐ Building C
3. Room number: ____________
4. Body temperature: ____________°C
5. Clinical symptoms (Please check all the symptoms you have.)
    - ☐ No symptom
    - ☐ Fever (≥37.5°C)
    - ☐ Coughing
    - ☐ Muscle pain
    - ☐ Runny nose or stuffy nose
    - ☐ Sore throat
    - ☐ Dyspnea
    - ☐ Feebleness (Malaise)
    - ☐ Diarrhea
    - ☐ Abdominal pain
    - ☐ Others (____________)
6. Do you have severe symptoms that need doctor's consultation?
    - ☐ Yes
    - ☐ No
7. Other requests about medical support: ____________

B. Gyeongju Center

1. Body temperature: ____________°C
2. Clinical symptoms (Please check all the symptoms you have.)
    - ☐ Coughing
    - ☐ Muscle pain
    - ☐ Runny nose or stuffy nose
    - ☐ Sore throat
    - ☐ Feebleness (Malaise)
    - ☐ Vomiting
    - ☐ Diarrhea
    - ☐ Abdominal pain
    - ☐ Others (____________)

C. Jecheon Center

1. Name: _____________
2. Location
   - □ Building B
   - □ Building C
3. Room number: _____________
4. Body temperature: _____________°C
5. Clinical symptoms (Please check all the symptoms you have.)
   - □ No symptom
   - □ Fever (≥37.5°C)
   - □ Coughing
   - □ Muscle pain
   - □ Runny nose or stuffy nose
   - □ Sore throat
   - □ Dyspnea
   - □ Feebleness (Malaise)
   - □ Diarrhea
   - □ Abdominal pain
   - □ Other (_____________)
6. Do you have severe symptoms that need doctor's consultation?
   - □ Yes
   - □ No
